# Treatment response and survival outcomes with BCR-ABL tyrosine kinase inhibitors in chronic myeloid leukemia: a retrospective study

**DOI:** 10.3389/fmed.2026.1905107

**Published:** 2026-08-19

**Authors:** Yufen Liu, Liang Zhang, Lu Zeng

**Affiliations:** 1 Department of Pharmacy, Ganzhou Hospital-Nanfang Hospital, Southern Medical University (Ganzhou People’s Hospital), Ganzhou, Jiangxi, China; 2 Department of Planning and Quality Control, Ganzhou Hospital-Nanfang Hospital, Southern Medical University (Ganzhou People’s Hospital), Ganzhou, Jiangxi, China

**Keywords:** BCR-ABL, chronic myeloid leukemia (CML), molecular response, overall survival, progression-free survival, tyrosine kinase inhibitors (TKI)

## Abstract

**Background:**

Chronic myeloid leukemia (CML) is a highly controllable cancer after the introduction of BCR-ABL tyrosine kinase inhibitors (TKIs). However, in real-world settings, there is limited evidence on treatment response, resistance, and survival.

**Objective:**

To measure treatment response, overall survival, and predictors of molecular response in patients with CML treated with BCR-ABL TKIs in routine clinical practice.

**Methodology:**

This was a retrospective cohort study consisting of 650 adult patients with confirmed CML who received at least one BCR-ABL TKI between January 2023 and December 2025. All demographic, clinical, treatment, and follow-up data were abstracted from the institution’s medical records. Assessment was performed for hematologic, cytogenetic, and molecular responses according to standard criteria. Overall survival (OS) and progression-free survival (PFS) were assessed using Kaplan–Meier analysis, and predictors of response and survival were identified using multivariable regression models.

**Results:**

The mean age was 49.8 ± 14.2 years, and 57.8% were male. The majority of patients were seen in the chronic phase (91.1%). Complete hematologic response, complete cytogenetic response, major molecular response (MMR) and deep molecular response (DMR) were observed in 93.4, 83.4, 76.3 and 48.9% of patients, respectively. The second-generation TKIs had a significantly higher MMR rate than imatinib (82.9% vs. 71.8%, *p* < 0.001). 17.4% of patients were treatment-resistant, and 14.9% were treatment-intolerant. Three-year OS and PFS were 95.8 and 93.4%. MMR was independently predicted by younger age, chronic-phase disease, lower ELTS risk and second-generation TKIs.

**Conclusion:**

Excellent molecular responses and favourable survival outcomes were observed among real-world patients with CML treated with BCR-ABL tyrosine kinase inhibitors. Molecular response remained an important prognostic indicator, supporting continued molecular monitoring and risk-adapted treatment. However, the available follow-up was insufficient to evaluate late-onset toxicities and longer-term treatment outcomes fully; therefore, extended follow-up studies with detailed molecular and genetic characterisation are warranted.

## 1 Introduction

Chronic myeloid leukaemia (CML) is a clonal myeloproliferative neoplasm characterised by the Philadelphia chromosome (BCR-ABL1), which encodes a constitutively active tyrosine kinase (1). Myeloid cells are overstimulated by persistent kinase activation, leading to uncontrolled proliferation, reduced apoptosis, and disease progression. The advent of BCR-ABL1 tyrosine kinase inhibitors (TKIs) completely changed CML from an almost invariably terminal illness to a largely controllable malignancy with extended survival (2). However, there is interpatient variability in treatment response, resistance, intolerance, disease course, and survival in real-world settings (3).

The use of BCR-ABL1 TKIs as the primary treatment for CML has established imatinib as the benchmark of targeted therapy, demonstrating their effectiveness. At the same time, second-generation TKIs offer treatment options for patients who are intolerant of, resistant to, or unresponsive to first-generation TKIs (4, 5). These treatments have significantly enhanced haematological, cytogenetic, and molecular responses; however, treatment responses can vary by disease phase, baseline risk, presence of other underlying illnesses, treatment line, and individual tolerance. Thus, the evaluation of treatment response and survival between treatment patterns with the different TKIs is clinically relevant (6).

Evaluation of treatment response remains a key component of CML management. Currently, the most common approach for assessing therapy effectiveness and disease extent is to measure BCR-ABL1 transcript levels quantitatively. The early molecular responses are associated with better long-term outcomes, and treatment response and survival may differ by transcript subtype (7). Treatment-related adverse events may necessitate dose adjustment or switching therapeutic agents (TKIs), which could affect treatment adherence and outcomes (8). Despite these advances, treatment outcomes observed in routine clinical practice may differ from those reported in clinical trials because of variations in patient characteristics, comorbidities, treatment adherence, resistance, and toxicity. Therefore, continued evaluation of treatment response, treatment modifications, and survival in real-world populations with CML remains clinically important (9).

However, while TKI therapy is effective, the outcomes of therapy in routine clinical practice may differ from those seen in controlled clinical trials, as patients in clinical practice often have comorbidities and variable disease risk, may be intolerant of treatment or have to adjust the dose, and may experience interruptions or switching to different therapies. Furthermore, the impact of baseline disease characteristics, TKI generation, treatment resistance, and molecular response on long-term survival is still clinically relevant. Hence, there is a need for real-world assessment of these factors for understanding the effectiveness and limitations of TKI therapy in a broader patient population. The present study aims to address this need by performing a combined analysis of the hematologic, cytogenetic, and molecular responses, treatment resistance and intolerance, adverse events, treatment changes, disease progression, survival, and independent predictors of major molecular response and overall survival in a large cohort of CML patients treated in routine clinical practice.

The main aim of this study was to assess treatment efficacy and survival among CML patients treated with BCR-ABL1 TKI therapy in clinical practice. The major endpoints are hematologic, cytogenetic, and molecular treatment response, including complete hematologic response (CHR), complete cytogenetic response (CCyR), major molecular response (MMR), deep molecular response (DMR), and overall survival (OS). The secondary endpoints were progression-free survival (PFS), treatment resistance, treatment intolerance, treatment modification and switching, treatment-related adverse events, and disease progression. Multivariable analyses were also carried out to identify independent predictors of MMR and OS, clinically and treatment-related. This study included treatment response, toxicity, treatment changes, and survival, to allow for a comprehensive evaluation of the effectiveness of TKIs in a real-world population of CML patients.

## 2 Methodology

### 2.1 Study design and setting

The goal of this retrospective cohort study was to assess treatment response and survival of patients with chronic myeloid leukaemia (CML) treated with BCR-ABL tyrosine kinase inhibitors (TKIs). It was a retrospective cohort study conducted at the Department of Haematology and Oncology, Ganzhou Hospital–Nanfang Hospital (Southern Medical University), in Ganzhou, Jiangxi, China, at a single centre. The study included adult patients with confirmed chronic myeloid leukaemia (CML) who initiated therapy with BCR-ABL1 tyrosine kinase inhibitors (TKIs) between January 2023 and December 2025. Clinical, laboratory, treatment, and follow-up information was collected by retrospective extraction from electronic medical records, laboratory databases, pharmacy records, and follow-up documentation.

Patients were followed from initiation of BCR-ABL tyrosine kinase inhibitor therapy until death, loss to follow-up, or the end of the study period (31 December 2025), whichever occurred first. The median follow-up duration was calculated for the study cohort. Treatment effectiveness; hematologic, cytogenetic, and molecular responses; treatment resistance and intolerance; and survival outcomes were assessed among patients receiving first-, second-, and third-line BCR-ABL tyrosine kinase inhibitors during the study period.

The available follow-up period allowed evaluation of hematologic, cytogenetic, and molecular responses, treatment modifications, frequent adverse events, and survival outcomes. The study was not designed to determine late-onset toxic effects or long-term treatment outcomes; however, treatment for CML is long-term, and some complications can present after prolonged exposure to TKIs.

### 2.2 Study population

The study population consisted of adult patients with CML who had received at least one BCR-ABL tyrosine kinase inhibitor (TKI) during the study period. Patients were identified from hospital cancer registries, haematology clinic databases, pharmacy dispensing records, and molecular diagnostic laboratory records.

**Figure 1:**
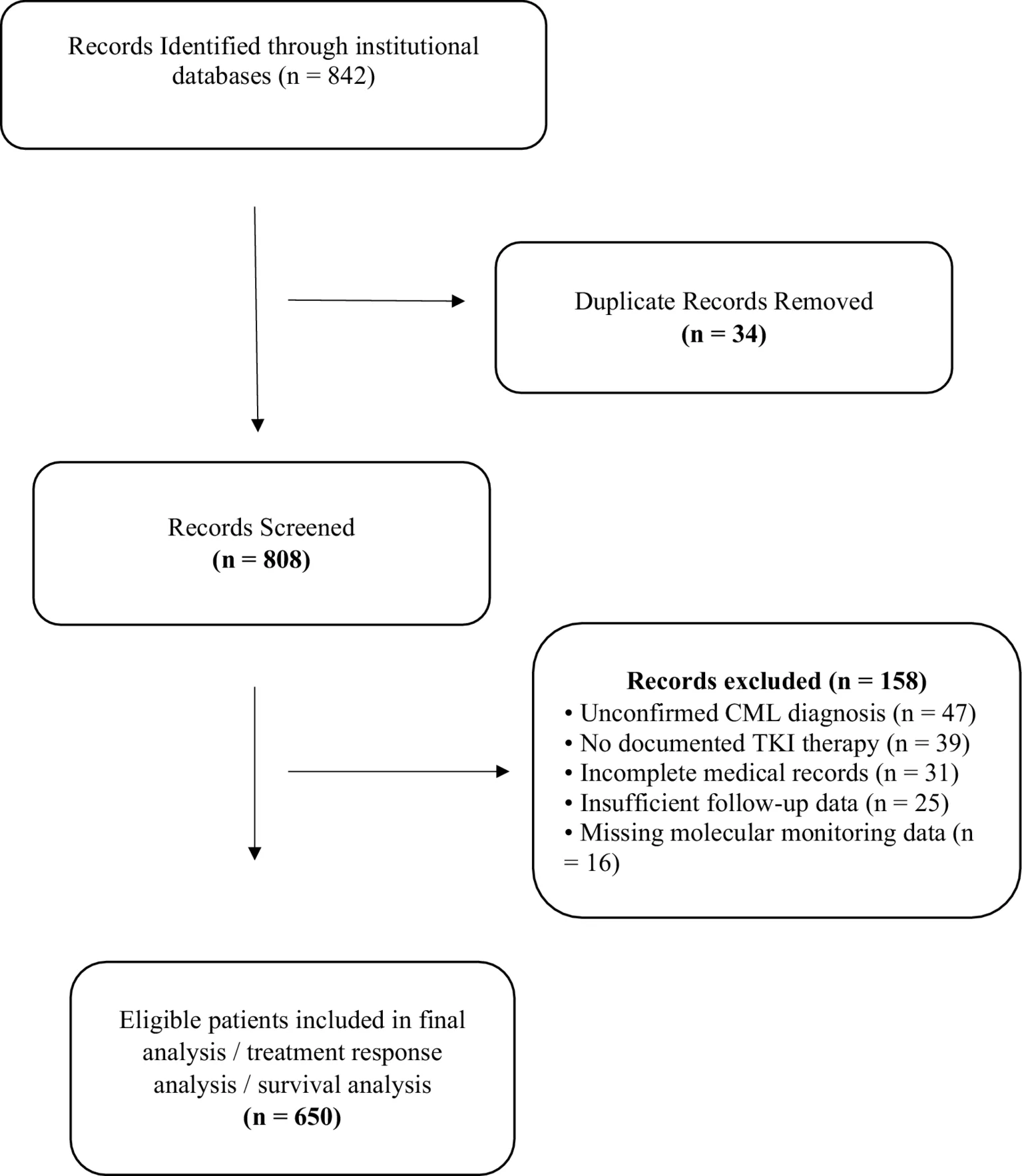
Participation flow diagram.

Initially, 842 patient records were found using the database screening process. After removing duplicate entries, patients without a confirmed diagnosis of CML, those not treated with TKI therapy, and records lacking information on treatment or follow-up were excluded. Other exclusions included patients with missing molecular monitoring data, an ambiguous treatment history, or loss to follow-up after treatment initiation. A total of 650 patients met all inclusion criteria and were analysed after applying all criteria (Figure 1).

Selection bias was minimised by including consecutive eligible patients, which provided a representative sample of clinical practice. A systematic screening procedure was used to identify patients. At first, all records found within the study period that might have been eligible were scanned. Haematological, cytogenetic, and molecular criteria were used to confirm the diagnosis of CML, and screening records were maintained. The patients were then evaluated for BCR-ABL TKI treatment and baseline and follow-up clinical information.

Of the records screened, those that did not meet diagnostic criteria, were duplicates, or lacked treatment documentation or adequate follow-up information were excluded. A total of 650 patients met all inclusion criteria and were included in the treatment response and survival analyses, constituting the final cohort.

### 2.3 Inclusion and exclusion criteria

The inclusion criteria were that patients in the study had a confirmed diagnosis of chronic myeloid leukaemia and a documented history of treatment with a BCR-ABL tyrosine kinase inhibitor, with sufficient follow-up data available during the study period. Baseline clinical data, treatment records, and follow-up assessments must be available to the patient to assess treatment response and survival outcomes.

Patients with incomplete medical records, an undetermined diagnosis, no documented exposure to TKIs, no follow-up data, or a missing key outcome variable were excluded. Patients enrolled in experimental treatment protocols without available outcome data were excluded.

### 2.4 Data collection procedures

The data were extracted using the standardised data-collection form designed for the study. Medical records, laboratory databases, pharmacy dispensing systems, and molecular monitoring reports were used as sources of information.

Baseline variables included age of diagnosis, sex, BMI, disease phase at diagnosis, comorbid conditions, baseline haematological parameters, spleen size, risk stratification scores (where applicable) and level of BCR-ABL transcripts. Treatment-related variables included: type of TKIs, line of therapy, treatment duration, treatment modifications, treatment interruption, treatment switching, timing of treatment switch, indication for switching (resistance, intolerance, disease progression, or other reasons), and clinical outcomes following treatment change. Molecular response was assessed using quantitative real-time PCR standardised to the International Scale (IS).

Institutional practice and international guidelines were followed for routine patient monitoring. Hematologic assessments were performed at baseline and every 2–4 weeks until a complete hematologic response, then every 3 months. Cytogenetic evaluation was routinely done at 6 and 12 months if clinically indicated. Patient follow-up was performed at approximately 3-month intervals using quantitative real-time polymerase chain reaction (qRT-PCR) to measure the BCR-ABL1 transcript on the International Scale (IS). The molecular assay was analytically sensitive enough to detect at least MR4 (BCR-ABL1 ≤ 0.01%) and thus the major and deep molecular responses could be assessed.

### 2.5 Key variables and operational definitions

#### 2.5.1 Chronic myeloid leukaemia

Chronic myeloid leukaemia was diagnosed based on the presence of the Philadelphia chromosome and/or detection of the BCR-ABL1 fusion transcript by cytogenetic analysis, fluorescence *in situ* hybridisation (FISH), or molecular testing.

#### 2.5.2 BCR-ABL tyrosine kinase inhibitor therapy

BCR-ABL tyrosine kinase inhibitor therapy was defined as receiving an FDA-approved targeted agent targeting BCR-ABL1 (first-generation or subsequent-generation TKI, per institutional treatment protocol).

#### 2.5.3 Complete hematologic response (CHR)

The criteria for complete hematologic response were defined as normalisation of peripheral blood counts, absence of immature granulocytes in the peripheral blood, disappearance of symptoms, and disappearance of palpable splenomegaly.

#### 2.5.4 Complete cytogenetic response (CCyR)

Complete cytogenetic response was defined as the absence of detectable Philadelphia chromosome-positive metaphase cells on cytogenetic evaluation.

#### 2.5.5 Major molecular response (MMR)

Major molecular response (MMR) was defined as BCR-ABL1 ≤ 0.1% on the International Scale (IS), corresponding to a ≥ 3-log reduction from the standardised baseline.

#### 2.5.6 Deep molecular response (DMR)

Deep molecular response (DMR) was defined as MR4 (BCR-ABL1 ≤ 0.01% IS) or more molecular remission by a standardized quantitative real-time PCR (qRT-PCR) assay with an analytical sensitivity of MR ≥ 0.01% IS.

#### 2.5.7 Treatment resistance

Treatment resistance was defined as failure to achieve expected hematologic, cytogenetic, or molecular response milestones, disease progression during therapy, or loss of a previously obtained response, all of which necessitated a change in therapy.

#### 2.5.8 Treatment intolerance

Treatment intolerance was considered a clinically relevant adverse event leading to a reduction of the prescribed TKI dose, temporary disruption or definitive withdrawal.

#### 2.5.9 Overall survival

Overall survival was defined as the time from initiation of TKI therapy to death from any cause or last follow-up.

#### 2.5.10 Progression-free survival

Progression-free survival was defined as the time from TKI initiation to progression to accelerated/blast phase or death from any cause.

### 2.6 Outcome measures

Primary outcomes included treatment response and overall survival. Treatment response included hematologic, cytogenetic and molecular response rates during follow-up. Secondary outcomes were progression-free survival, treatment resistance, treatment intolerance, therapy switching patterns, and treatment or drug resistance associated with favourable or unfavourable clinical outcomes.

### 2.7 Statistical analysis

The statistical analysis was done using SPSS Statistics version 27.0 (IBM Corp., Armonk, NY, USA). Continuous variables were assessed for normality and reported as mean ± SD or median (IQR), depending on which was more appropriate. Frequency and percentages were used to summarize categorical variables.

Independent-samples *t*-tests, Mann–Whitney U tests, chi-square tests, or Fisher’s exact tests were used to compare the treatment groups, depending on the data. The Kaplan–Meier method was used to estimate survival, and the log-rank test to compare survival curves.

Initially, univariable analyses were used to determine factors associated with treatment response and survival outcomes. Variables with *p* < 0.10 in univariable analyses or those considered clinically relevant based on prior literature were entered into multivariable models. Hazard ratios and 95% confidence intervals (CIs) were computed. A *p*-value < 0.05 in both directions was deemed statistically significant.

### 2.8 Quality control and data validation

Trained investigators independently verified all records obtained to ensure the accuracy and reliability of the data. Data cleaning activities included consistency checks, validation of treatment dates, and verification of response evaluation dates and survival status. Data discrepancies found during the data review were addressed through re-examination of source documents and consensus among investigators.

### 2.9 Ethical considerations

This research was approved by the Ethics Committee of Ganzhou Hospital - Nanfang Hospital, Approval Number: 2024-474-01. The study was carried out in accordance with the ethical guidelines of the Declaration of Helsinki and its subsequent amendments regarding research involving human subjects.

## 3 Results

### 3.1 Baseline demographic and clinical characteristics

Finally, 650 patients with chronic myeloid leukaemia (CML) who met the inclusion criteria were analysed. After application of the predefined exclusion criteria, complete data were available for all 650 included patients, and no additional missing data were identified for the variables included in the primary analyses. Demographic and clinical baseline data are reported in Table 1. The age at diagnosis was 49.8 ± 14.2 years, and the largest proportion of patients was in the 41–60 age group (46.3%). The overall male-to-female ratio was about 1.4:1, with 57.8% of patients male. The mean body mass index was 26.3 ± 4.8 kg/m^2^.

**Table 1:** Baseline demographic and clinical characteristics of the study population (*N* = 650).

Variable	*n* (%) or Mean ± SD
Age at diagnosis (years)	49.8 ± 14.2
≤40 years	198 (30.5)
41–60 years	301 (46.3)
>60 years	151 (23.2)
Male	376 (57.8)
Female	274 (42.2)
BMI (kg/m^2^)	26.3 ± 4.8
Current smoker	117 (18.0)
Hypertension	162 (24.9)
Diabetes mellitus	128 (19.7)
Cardiovascular disease	71 (10.9)
Chronic kidney disease	34 (5.2)
Charlson Comorbidity Index ≥2	138 (21.2)
Splenomegaly at diagnosis	401 (61.7)
ECOG Performance Status 0–1	572 (88.0)
ECOG Performance Status ≥2	78 (12.0)

Values are presented as mean ± standard deviation or frequency (percentage). BMI, body mass index; ECOG, Eastern Cooperative Oncology Group.

Most patients had comorbidities at baseline, including hypertension (24.9%), diabetes mellitus (19.7%), cardiovascular disease (10.9%), and chronic kidney disease (5.2%). Charlson Comorbidity Index ≥2 was documented in 21.2% of the patients. At diagnosis, 61.7% of patients were splenomegalic, and the majority had a good performance status (ECOG score 0–1; Table 1).

### 3.2 Disease characteristics at diagnosis

Table 2 summarizes the baseline information of the diseases. Most patients had been diagnosed in the chronic phase of the disease (91.1%), with 6.3 and 2.6% diagnosed in the accelerated and blast phases, respectively. 34.5% of patients were classified as low risk, 42.0% as intermediate risk, and 23.5% as high risk (according to Sokal risk stratification). The same distribution was observed using the ELTS scoring system, with 37.8, 42.5, and 19.7% in the low-, intermediate-, and high-risk groups, respectively. All patients had molecular evidence of disease (BCR-ABL1 transcripts), with a median white blood cell count at diagnosis of 89.4 × 10^9^/L and a median platelet count of 468 × 10^9^/L.

**Table 2:** Baseline disease characteristics.

Variable	*n* (%)
Chronic phase	592 (91.1)
Accelerated phase	41 (6.3)
Blast phase	17 (2.6)
Low Sokal risk	224 (34.5)
Intermediate Sokal risk	273 (42.0)
High Sokal risk	153 (23.5)
Low ELTS risk	246 (37.8)
Intermediate ELTS risk	276 (42.5)
High ELTS risk	128 (19.7)
Median WBC count (×10^9^/L)	89.4 (IQR 52.7–136.5)
Median platelet count (×10^9^/L)	468 (IQR 312–681)
BCR-ABL transcript detectable	650 (100)
Baseline haemoglobin (g/dL), mean ± SD	10.4 ± 2.1

Sokal and ELTS scores were calculated using baseline clinical and hematologic parameters.

### 3.3 Treatment characteristics

In summary, the treatment patterns are presented in Table 3. The most commonly used first-line TKI was imatinib, used by 358 patients (55.1%), followed by nilotinib (22.2%), dasatinib (19.4%) and bosutinib (3.3%). At follow-up, 504 patients (77.5%) continued on the initial TKI therapy, 118 (18.2%) moved to the second line, and 28 (4.3%) to the third line. 21.1% of patients required dose modification, 17.2% had temporary interruption of treatment, and 9.1% had permanent discontinuation. In all, 22.5% of patients experienced a change in treatment. Treatment exposure during the observation period was high, with a median TKI therapy duration of 24.7 months (IQR: 16.2–32.1). Resistance and intolerance were the most common reasons for switching therapy among 146 patients who did so during follow-up. Supplementary Table S1 lists the details of switching treatments and the choice of subsequent TKIs.

**Table 3:** Treatment patterns and TKI utilization.

Variable	*n* (%)
First-line TKI therapy	
Imatinib	358 (55.1)
Nilotinib	144 (22.2)
Dasatinib	126 (19.4)
Bosutinib	22 (3.3)
Line of therapy during follow-up	
Remained on first-line therapy	504 (77.5)
Required second-line therapy	118 (18.2)
Required third-line therapy	28 (4.3)
Treatment management	
Dose modification required	137 (21.1)
Temporary treatment interruption	112 (17.2)
Permanent discontinuation	59 (9.1)
Treatment switch during follow-up	146 (22.5)
Median time to first treatment switch (months)	13.4 (8.1–19.7)
Switch to second-line therapy	118 (18.2)
Switch to third-line therapy	28 (4.3)
Treatment duration	
Median treatment duration, months (IQR)	24.7 (16.2–32.1)

Treatment categories indicate exposure to BCR-ABL tyrosine kinase inhibitors during the study period. Percentages are calculated using the total study population (*N* = 650). IQR, interquartile range.

In follow-up, 146 patients (22.5%) needed a change in treatment following a median of 13.4 months (IQR 8.1–19.7) from treatment initiation. The most common indications for changing therapy were drug intolerance (36.3%) and treatment resistance (primary resistance, 32.9%; secondary resistance/loss of response, 21.2%). The majority of patients were then treated with nilotinib or dasatinib, while fewer received subsequent next–generation TKIs such as bosutinib or ponatinib. After treatment modification, about 75% of switched patients had a complete cytogenetic response, 2/3 had a major molecular response, and more than one-third had a deep molecular response, with a small group demonstrating ongoing resistance and necessitating continued treatment escalation (Supplementary Table S1).

### 3.4 Hematologic, cytogenetic, and molecular responses

Treatment response was observed early after initiation of TKI treatment, as shown in Table 4. A complete hematologic response was observed in 607 patients (93.4%), with a median time to response of 1.8 months (IQR 1.2–2.9). A median of 8.6 months after treatment, 542 patients (83.4%) had a complete cytogenetic response, and 496 (76.3%) had a major molecular response. With follow-up molecular response (FMR), progressive depth of molecular remission was observed, with deep molecular response (deepMR) achieved in 318 patients (48.9%) at a median of 21.8 months. MMR remained sustained in 442 patients (68.0%), but only 54 patients (8.3%) lost molecular response, which was sustained over a long follow-up period of 26.4 months (median). Most patients who showed MMR had durable disease control.

**Table 4:** Hematologic, cytogenetic, and molecular response outcomes during follow-up (*N* = 650).

Response outcome	Patients achieving response *n* (%)	Median time to response, months (IQR)
Complete Hematologic Response (CHR)	607 (93.4)	1.8 (1.2–2.9)
Complete Cytogenetic Response (CCyR)	542 (83.4)	8.6 (6.4–11.2)
Major Molecular Response (MMR)	496 (76.3)	13.1 (10.2–17.5)
Deep Molecular Response (DMR)	318 (48.9)	21.8 (17.4–28.6)
Sustained MMR	442 (68.0)	Not applicable
Loss of molecular response	54 (8.3)	Median time to loss: 26.4 (19.5–34.1)

Responses represent the best documented response achieved during follow-up. Median time to response was calculated from initiation of BCR-ABL tyrosine kinase inhibitor therapy to the first documented achievement of each response milestone. CHR, Complete Hematologic Response; CCyR, Complete Cytogenetic Response; MMR, Major Molecular Response (BCR-ABL1 ≤ 0.1% IS); DMR, Deep Molecular Response (MR4 or deeper).

Overall treatment responses were favorable, with 93.4% of patients achieving CHR and 83.4% achieving CCyR during follow-up. Major molecular response was achieved in 76.3% of patients, and nearly half achieved DMR, indicating substantial disease burden reduction with TKI therapy. Sustained MMR was maintained in most responders, whereas loss of molecular response occurred infrequently, reflecting durable treatment effectiveness in routine clinical practice.

### 3.5 Comparison of response according to initial TKI

The distribution of responses by first-line TKI exposure is presented in Table 5. Patients treated with second-generation TKIs had significantly higher response rates across all response categories compared with those treated with imatinib. A complete hematologic response was obtained in 279/291 patients (95.8%) treated with second-generation TKIs versus 328/358 patients (91.6%) treated with imatinib (*p* = 0.041). Likewise, the second-generation TKI group had a significantly higher CCyR rate than the first-generation TKI group (87.6% vs. 80.7%, *p* = 0.018). Significant differences were observed in molecular outcomes, with higher rates among patients treated with second-generation TKIs than among those treated with imatinib (82.9% vs. 71.8%, *p* < 0.001; 57.8% vs. 42.5%, *p* < 0.001; Table 5).

**Table 5:** Molecular response according to first-line TKI.

Outcome	Imatinib	2G TKIs*	*p*-value
CHR (%)	91.6	95.8	0.041
CCyR (%)	80.7	87.6	0.018
MMR (%)	71.8	82.9	<0.001
DMR (%)	42.5	57.8	<0.001

*Nilotinib, Dasatinib, Bosutinib.

Baseline characteristics stratified by initial treatment group are presented in Supplementary Table S2. Patients receiving second-generation TKIs had modest differences in baseline disease risk compared with those receiving imatinib; therefore, comparisons between treatment groups should be interpreted with caution, as treatment allocation was based on clinical decision-making rather than randomisation.

### 3.6 Treatment resistance and intolerance

The outcomes of treatment resistance and treatment intolerance are reported in Table 6. Overall, 9.4% of patients were primary resistant, and 8.0% had secondary resistance, for a total of 113 patients (17.4%). The incidence of drug intolerance was recorded in 14.9% of patients.

**Table 6:** Resistance and intolerance outcomes.

Variable	*n* (%)
Primary resistance	61 (9.4)
Secondary resistance	52 (8.0)
Any resistance	113 (17.4)
Drug intolerance	97 (14.9)
Therapy switched due to resistance	79 (12.2)
Therapy switched due to intolerance	53 (8.2)

Of those who needed therapeutic changes, 12.2% switched therapy due to resistance and 8.2% due to intolerance. Overall, these results underscore the ongoing clinical relevance of resistance monitoring and toxicity management in contemporary TKI therapy (Table 6).

Treatment intolerance accounted for more than 1/3 of therapy switches, and primary and secondary resistance combined accounted for more than 1/2 of therapy switches (as detailed in Supplementary Table S1). After therapy modification, second-generation TKIs were the most common choice for subsequent treatment, while ponatinib was used primarily for those with more refractory disease courses.

### 3.7 Adverse events

The AE profiles differed across drugs, depending on which TKI was administered as first-line therapy (Table 7). Fluid retention was more common in patients treated with imatinib, while pleural effusion was more common in patients treated with dasatinib. Gastrointestinal toxicity was more common in all treatment groups, whereas hepatic toxicity and cardiovascular events were relatively more common in nilotinib-treated patients. However, the incidence of grade 3–4 toxicity did not differ significantly and permanent discontinuation of treatment because of toxicity was rare in all TKIs. These findings represent adverse events documented during the available follow-up period. They should not be interpreted as a complete assessment of late-onset or cumulative toxicities associated with prolonged TKI exposure.

**Table 7:** Treatment-related adverse events according to first-line tyrosine kinase inhibitor.

Adverse event	Imatinib *n* (%)	Nilotinib *n* (%)	Dasatinib *n* (%)	Bosutinib *n* (%)	p-value
Hematologic toxicity	59	23	25	5	0.37
Fluid retention	48	9	13	4	0.011
Gastrointestinal toxicity	44	18	23	12	0.043
Hepatic toxicity	17	18	10	8	0.028
Cardiovascular events	8	14	6	3	0.031
Pleural effusion	2	3	21	2	<0.001
Grade 3–4 toxicity	18	11	13	4	0.46
Permanent discontinuation	25	12	17	5	0.55

### 3.8 Disease progression and survival outcomes

The clinical results of follow-up are summarized in Table 8. Disease progression was rare; 21 patients (3.2%) progressed to accelerated phase, and 2.2% to blast phase. There was a 2.9% mortality due to disease. 38 deaths were recorded during the follow-up, giving an overall mortality rate of 5.8% at the end of follow up. Overall, the disease control and survival were good, with 94.2% of patients still alive at the last follow-up point (Table 8).

**Table 8:** Clinical outcomes during follow-up.

Outcome	*n* (%)
Progression to accelerated phase	21 (3.2)
Progression to blast phase	14 (2.2)
Disease-related deaths	19 (2.9)
Deaths from any cause	38 (5.8)
Alive at last follow-up	612 (94.2)
Median follow-up, months (IQR)	24.7 (16.2–32.1)

### 3.9 Kaplan–Meier survival analysis

**Figure 2:**
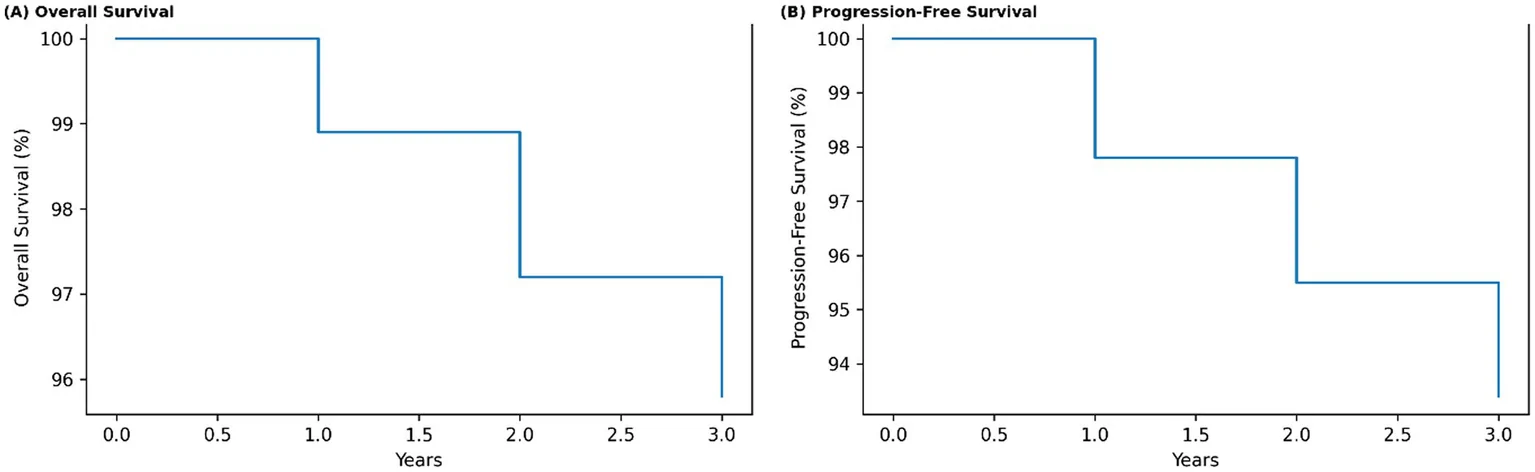
Kaplan–Meier survival analyses of overall survival and progression-free survival during the 3-year follow-up period. **(A)** Overall survival (OS) and **(B)** Progression-free survival (PFS) among study participants. Survival probabilities were estimated over time, with the proportion of surviving patients displayed on the y-axis and follow-up duration (years) on the x-axis.

The Kaplan–Meier survival analysis showed very good results for the entire follow-up period (Table 9). The estimated overall survival rates at 1 year and 3 years were 98.9 and 95.8%, respectively. Likewise, progression-free survival at 1 and 3 years was 97.8 and 93.4%, respectively. Kaplan–Meier curves were visually analyzed for evidence of survival probabilities that remained stable over time (Figure 2). Patients who achieved a Major Molecular Response had significantly better PFS than those who did not achieve molecular milestones.

**Table 9:** Survival estimates.

Survival measure	Estimate (%)	95% CI
1-year Overall Survival	98.9	97.9–99.8
3-year Overall Survival	95.8	93.8–97.8
1-year Progression-Free Survival	97.8	96.5–99.1
3-year Progression-Free Survival	93.4	90.8–96.0

### 3.10 Predictors of major molecular response

Several independent predictors of achieving a major molecular response were found by multivariable logistic regression analysis (Table 10). Patients younger than 60 years had significantly higher odds of attaining MMR (OR = 1.74, 95% CI: 1.18–2.56, *p* = 0.005). Likewise, low/intermediate ELTS risk status at diagnosis (OR = 2.11, *p* < 0.001), chronic-phase disease at diagnosis (OR = 2.68, *p* = 0.002) and treatment with second-generation TKIs (OR = 1.92, *p* = 0.001) were all significantly associated with better molecular response rates. However, a Charlson Comorbidity Index score >2 was associated with lower odds of a successful MMR result (OR = 0.71, *p* = 0.034).

**Table 10:** Multivariable logistic regression analysis.

Variable	Adjusted OR	95% CI	*p*-value
Age <60 years	1.74	1.18–2.56	0.005
Low/Intermediate ELTS risk	2.11	1.39–3.19	<0.001
Chronic phase disease	2.68	1.41–5.07	0.002
Second-generation TKI	1.92	1.31–2.83	0.001
Charlson Index ≥2	0.71	0.50–0.97	0.034

OR, Odds Ratio; CI, Confidence Interval.

### 3.11 Predictors of overall survival

Results of the multivariable Cox proportional hazards model are shown in Table 11. Overall survival was significantly poorer in those >60 years old (HR = 2.84, 95% CI: 1.54–5.23, *p* = 0.001) independently. Likewise, high ELTS risk (HR = 2.49, *p* = 0.004), treatment resistance (HR = 2.97, *p* < 0.001), and failure to achieve MMR (HR = 3.76, *p* < 0.001) significantly increased mortality risk.

**Table 11:** Multivariable Cox proportional hazards model for overall survival.

Variable	HR	95% CI	*p*-value
Age >60 years	2.84	1.54–5.23	0.001
High ELTS risk	2.49	1.33–4.65	0.004
Failure to achieve MMR	3.76	2.01–7.02	<0.001
Treatment resistance	2.97	1.61–5.47	<0.001
Second-generation TKI	0.72	0.42–1.23	0.228

HR, Hazard Ratio; CI, Confidence Interval.

The hazard for death was lower with second-generation TKI therapy (HR = 0.72), but this was not statistically significant after multivariable adjustment (*p* = 0.228), implying that disease biology and treatment response were more important for survival than initial TKI selection (Table 11).

**Figure 3:**
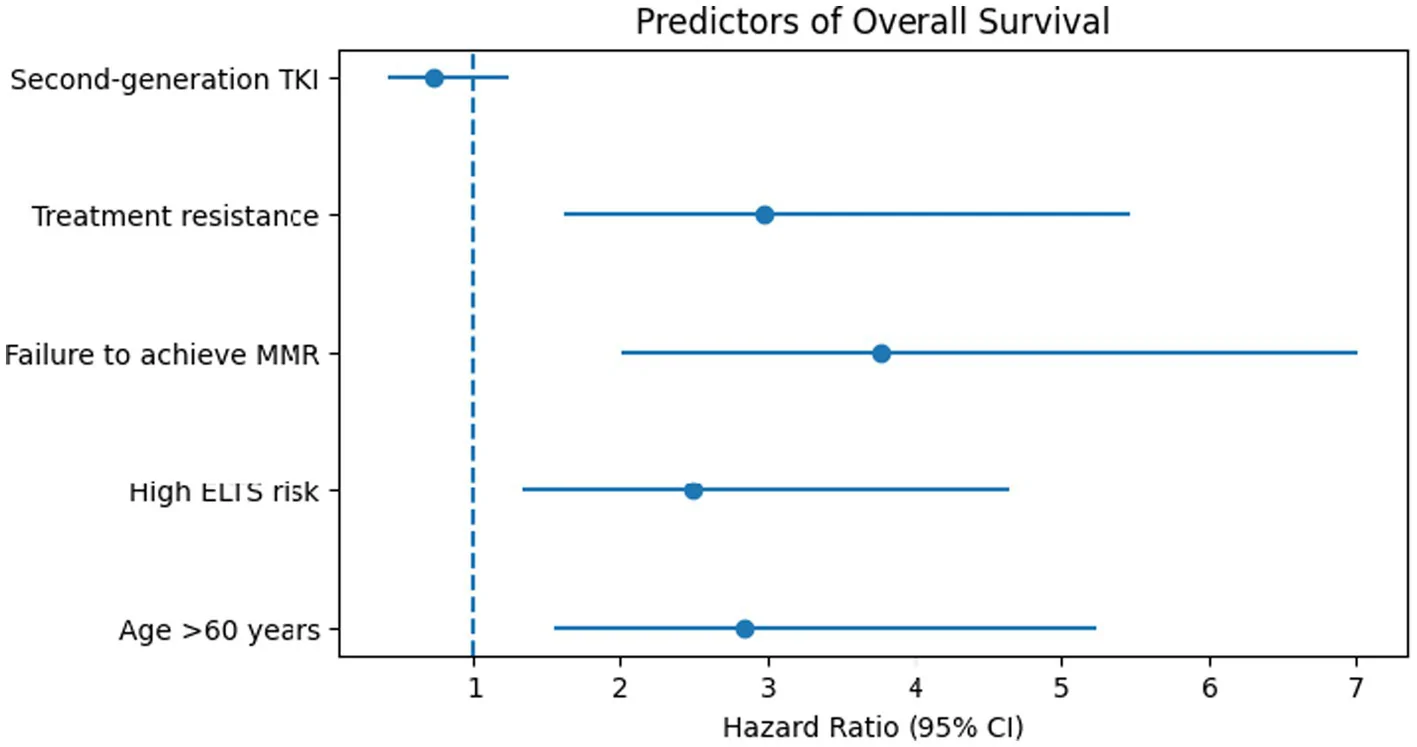
Multivariable Cox proportional hazards analysis of factors associated with overall survival in patients with chronic myeloid leukemia treated with BCR-ABL tyrosine kinase inhibitors. Forest plot illustrating hazard ratios (*HRs*) and 95% confidence intervals (*CIs*) for independent predictors of overall survival. Variables included age >60 years, high ELTS risk score, failure to achieve major molecular response (MMR), treatment resistance, and receipt of second-generation tyrosine kinase inhibitors (TKIs). Hazard ratios >1 indicate an increased risk of mortality, whereas hazard ratios <1 indicate a reduced risk of mortality. The vertical dashed line represents the null value (*HR* = 1.0).

The adjusted effects of independent prognostic factors on overall survival are presented in the forest plot shown in Figure 3.

## 4 Discussion

In the present study, patients who received BCR-ABL tyrosine kinase inhibitors had good baseline characteristics and overall treatment outcomes. The majority of patients were diagnosed during the chronic phase, and the mean age of 49.8 years was similar to that of other modern observational cohorts of patients with CML (10). However, there was a slight male predominance. Although the hypertension, diabetes mellitus and cardiovascular disease prevalence rates were relatively high, treatment outcomes were good, and survival rates were satisfactory across the cohort. These results are consistent with the growing body of evidence demonstrating that TKI treatment has dramatically improved long-term disease control and has resulted in durable molecular responses in routine clinical practice, potentially allowing treatment-free remission in the right patients (11, 12). Additionally, our findings are consistent with previous clinical experience that baseline comorbidities are more likely to affect treatment choice, long-term tolerability, and toxicity management than to impact the effectiveness of TKI therapy, and that these effects can be minimised with appropriate individualised therapeutic strategies. The patient is well-evaluated at follow-up (13).

The present cohort also provides real-world evidence in a tertiary care setting in China, and patients with clinically significant comorbidities such as hypertension, diabetes mellitus, cardiovascular disease and chronic kidney disease. This data could be used in conjunction with data from other geographic areas since patient characteristics, the presence of comorbidities, access to healthcare and treatment practices can differ between populations. As it was a single-centre study and no formal comparisons were made between racial and ethnic groups, the results should be understood as a population-based real-world experience rather than a reflection of racial differences.

In our cohort, the majority of patients were diagnosed in the chronic phase, and low- and intermediate-risk predominated on both the Sokal and ELTS scores. This distribution may have influenced the positive treatment responses and survival rates seen in follow-up. As in previous real-world ELTS studies, patients with lower ELTS risk had better molecular and survival outcomes, and this remained true in routine clinical use, reinforcing the value of ELTS for risk stratification (14). Moreover, our results provide evidence that ELTS is a valid prognostic discriminator in the TKI era and will help identify patients who might benefit from closer follow-up and tailored treatment compared with traditional risk models (15).

Imatinib was the most commonly used initial therapy, with second-generation TKIs (nilotinib, dasatinib, bosutinib) also used. This distribution is a mix of cost-driven prescribing and a preference for second-generation TKIs in high-risk disease, as guided by guidelines (16, 17). Furthermore, Held and Atallah (18) noted that despite the widespread introduction of second-generation therapies, with greater molecular response kinetics, imatinib remains the predominant therapy in many situations. The 77.5% who continued on first-line therapy demonstrate fairly good disease control, despite the 22.5% switching rate, indicating ongoing treatment difficulties in the long-term management of these patients. Chronic toxicity burden of lifelong TKI therapy is further underscored by dose modifications, interruptions and discontinuations, especially for those with comorbid conditions or complications due to intolerance.

In the follow-up period, around one-fourth of patients (12.5%) needed treatment switches and a median time to first switch of around 13 months. The main indication for switching treatment was drug intolerance and drug resistance, while disease progression and physician or patient preference played a minor role in the decision to switch treatment. The majority of patients went on to receive second-generation TKIs, mainly nilotinib and dasatinib. After treatment modification, almost three-quarters of patients had a complete cytogenetic response, two-thirds had a major molecular response, and more than one-third had a deep molecular response, with only a minority having persistent resistance and requiring further therapeutic escalation (Supplementary Table S1). This data confirms the efficacy of sequential BCR-ABL TKI therapy when first-line treatment fails or is intolerable, and reflects current real-world data showing benefit from timely therapeutic switching.

Excellent disease control, with high rates of complete hematologic response (93.4%), complete cytogenetic response (83.4%), and major molecular response (76.3%), is consistent with the real-world effectiveness of TKIs. In line with the results of Geelen et al. (19), who obtained similar response rates in population-based cohorts, the results from the clinical trials are replicable in routine clinical practice. A clinically relevant deep molecular response was observed in almost half of the patients, the biological landmark to qualify for treatment-free remission. Furthermore, real-world data support the growing feasibility of remission strategies that do not involve treatment in clinical care, provided they achieve sustained molecular remission over the long term (20, 21). Deep molecular responses are especially significant, as they are needed to implement treatment-free remission approaches, whereby certain patients can safely stop therapy in carefully monitored molecular remission (22). After 68% of patients had sustained MMR (durable disease suppression), the subset of patients without molecular suppression emphasises the dynamic nature of disease control, which depends on adherence, pharmacokinetics, and clonal evolution (23).

The median time to response also followed the expected therapeutic sequence, with hematologic remission occurring within the first few months of treatment, cytogenetic remission within the first year, and deep molecular responses developing later during continued TKI therapy. These findings are consistent with established response kinetics reported in contemporary real-world and observational CML cohorts.

The success rates of hematologic, cytogenetic, and molecular responses were significantly higher in patients receiving second-generation TKIs than in those receiving imatinib. This includes increased MMR and DMR, which indicate greater and quicker molecular repression. In line with this, Claudiani et al. (24) showed that second-generation TKIs result in earlier and deeper molecular response, potentially leading to better long-term treatment-free remission eligibility. As demonstrated by Mauro et al. (10), these higher response rates do not necessarily translate into a survival benefit, likely because of the availability of effective salvage therapies. These results support the idea that second-generation TKIs do not significantly alter survival, but rather increase the depth and rapidity of response if long-term management is optimised. However, because treatment allocation was not randomized, differences in baseline disease characteristics may have contributed to the observed treatment responses. Therefore, these comparisons should be interpreted as hypothesis-generating rather than causal.

Long-term therapy with TKIs is difficult, with an overall resistance rate of 17.4% and an intolerance rate of 14.9% among our patients. The most common treatment changes were due to resistance and drug intolerance, underscoring its ongoing role in long-term disease management in day-to-day clinical practice. This has been observed in real-world studies as well, with treatment decisions mostly influenced by lack of therapeutic response and adverse events rather than disease progression (25). The majority of patients with resistance or intolerance in our cohort achieved a favourable cytogenetic and molecular response to subsequent therapy change, indicating a clinical benefit of timely therapy change. The findings highlight the need for regular monitoring of responses and individualised treatment strategies to maximise long-term outcomes (26).

The safety profile seen was consistent with the known TKI toxicity profile. Hematologic toxicity was most common, followed by gastrointestinal, fluid retention, and hepatic toxicities. The cardiovascular events and the few cases of pleural effusion are also clinically relevant but less common. The authors of the study by Yoshifuji and Sasaki (8) noted that long-term toxicity monitoring is important during chronic TKI therapy, as toxic effects are often dose-dependent. Iurlo et al. (5) also noted that dose optimisation strategies are currently crucial to maximising a drug’s effectiveness and safety. This relatively low incidence of grade 3–4 toxicity (7.1%) indicates that most side effects were manageable, reinforcing the favourable risk–benefit ratio of TKIs in routine clinical practice. The observed toxicity profiles were consistent with the recognised safety profiles of individual TKIs, underscoring the importance of selecting therapy based on both disease risk and patient comorbidities.

The overall safety profile was relatively good, although a median follow-up period of 24.7 months is insufficient to assess late and cumulative toxicities of long-term TKI use. Some clinically relevant adverse effects may be more pronounced with greater exposure to treatment, such as cardiovascular, vascular, metabolic, and pulmonary effects. Thus, the safety results from the present study can be regarded as a reflection of the adverse-event profile during the follow-up period rather than long-term safety. Ongoing clinical and laboratory monitoring is important, especially for those with underlying medical conditions.

Progressive disease to accelerated or blast phase was infrequent, and disease deaths were low. This overall survival rate of 94.2% illustrates the breakthrough that TKIs have made in the treatment of CML, turning it into a chronic, manageable condition. Collins et al. (9) also showed that real-world survival data are now nearly identical to those from clinical trials. However, survival differences between groups persist due to both differences in the diseases present and variations in adherence. Notably, some deaths were not attributable to CML progression, emphasising the emerging role of competing risks in long-term survival analyses. The authors of the paper, Bourne et al. (22), highlighted the importance of sustained molecular response, which is a crucial factor for long-term survival and eligibility for treatment-free remission.

Well, the 1- and 3-year overall survival and progression-free survival rates are still very good in this group of patients. The findings are consistent with international clinical data, including those reported by Geelen et al. (19) and Sanz et al. (27), which also show promising survival trends during TKI treatment. Notably, patients with MMR showed significantly improved progression-free survival, underscoring the prognostic value of molecular response milestones. In a similar study, Mauro et al. (10) demonstrated a strong association between early molecular response and long-term outcomes, but this relationship may be attenuated after multivariable adjustment.

Younger age, low/intermediate ELTS risk, chronic-phase disease, and second-generation TKI therapy were independent predictors of achieving MMR in the multivariable analysis. These findings are plausible and consistent with previous studies. Saifullah and Lucas (28) emphasised that younger patients with a lower disease burden are more likely to have sustained deep molecular responses. Likewise, Stuckey et al. (29) noted that treatment intensity, in addition to disease biology, influences the probability of molecular remission. In contrast, an increased burden of comorbidities adversely affected the molecular response, which may be related to decreased therapy intensity, therapy disruption, or altered drug metabolism.

Poor overall survival was significantly associated with the following factors: age >60 years, high ELTS risk, treatment resistance and failure to attain MMR. The results underscore the importance of the interaction between disease biology and treatment response in predicting long-term outcomes. Iezza et al. (30) pointed out that dynamic risk models that account for molecular response outperformed models that use baseline variables. In addition, Bruzzese et al. (31) again highlighted the importance of achieving a deep molecular response, not just for survival but also for qualification for treatment-free remission therapies. Importantly, after adjustment, survival was not an independent predictor of second-generation TKI therapy, indicating that its benefit is largely driven by molecular response rather than direct survival benefit (32).

### 4.1 Strengths of the study

This study has several important strengths. The relatively large number of patients (650) allowed for robust statistical power and good treatment response, treatment resistance, treatment intolerance, adverse event, and prognostic factor analysis of the treatment group in a real-world setting of chronic myelogenous leukaemia (CML). The large sample size enabled assessment of patients across a wide range of demographic, disease presentation, comorbidity, and treatment characteristics. Secondly, the study reflects real-world clinical practice, where patients with diverse disease characteristics, comorbidities, treatment changes, and treatment adherence are often not represented in randomised clinical trials. Third, several clinically relevant outcomes were assessed simultaneously, including hematologic response, cytogenetic response, molecular response, treatment resistance, treatment intolerance, adverse events, progression, and survival. Fourth, the use of multivariable logistic and Cox regression analyses enabled identification of independent predictors of molecular response and overall survival. Lastly, the study provides up-to-date information on the comparative effectiveness of first- and second-generation TKIs, which can inform risk-adapted treatment decisions in clinical practice.

### 4.2 Limitations of the study

The results have to be interpreted with some limitations in mind. There is a risk of selection bias, information bias, and residual confounding with the retrospective design. The data were sourced from available medical records, but may be incomplete or inaccurate even after quality control. Because this was a single-centre retrospective study conducted at a tertiary care institution, the findings may not be fully generalisable to other healthcare settings or patient populations. The median follow-up of around 24.7 months was adequate to analyse their early and intermediate treatment responses and common side effects, but was relatively short to assess late adverse effects, cumulative complications of long-term TKI exposure, long-term treatment durability, the possibility of treatment-free remission, and very long-term survival. Hence, these good safety and survival results must not be taken as definitive proof of long-term safety and efficacy of TKIs. Follow-up is needed to define the long-term side effects and permanence of treatment effects. Because major molecular response was achieved during follow-up, its inclusion in survival modelling may be susceptible to immortal time bias. Future studies using time-dependent Cox regression or landmark analyses are warranted to further validate these findings.

Furthermore, detailed genetic and molecular information was not available in all cases. In particular, the analyses of comprehensive BCR-ABL1 kinase domain mutation analysis, other cytogenetic abnormalities, transcript subtype, clonal evolution, and wider genome profiling were not included in the primary analyses. Such factors could have an impact on TKI resistance, treatment choice, disease course, and prognosis. Furthermore, data on the adherence to treatment, socioeconomic factors, and patient-reported quality of life was not available. This is an observational study, and no causal inferences can be made regarding treatment strategies and outcomes. In this study, patients with chronic-, accelerated-, and blast-phase CML were included in primary analyses, while most published cohorts included for comparison were limited to chronic-phase disease. Patients with advanced disease made up only a minority of the study population, but may have limited the homogeneity of improvement rates and survival in our study. Thus, direct comparison of our results with other studies focusing on the chronic-phase CML should be interpreted with caution, and further studies with phase-specific analyses are required to confirm our observations.

### 4.3 Future recommendations

Studies with larger numbers of centres and longer follow-up periods are warranted to confirm these results and enhance external generalizability. Long-term follow-up for late-onset and cumulative toxicities of TKI should be performed in future longitudinal studies, especially cardiovascular and vascular, metabolic, pulmonary, hepatic and renal complications. In addition, in-depth molecular and genetic assays should be performed, such as BCR-ABL1 kinase domain mutation testing, transcript subtype testing, detection of other cytogenetic abnormalities, and, whenever possible, more comprehensive genomic testing. Combining these molecular data with clinical characteristics, comorbidity burden, treatment exposure, and molecular response will help accurately predict treatment resistance and select more individualised TKIs. Future studies should include molecular mutation profiling, adherence assessment, and patient-reported quality-of-life measures to identify the factors that determine long-term treatment success. Comparative effectiveness studies are also being conducted on newer therapeutic agents, such as asciminib and other targeted therapies. Moreover, future studies should identify predictors of deep molecular response and treatment-free remission, which have emerged as increasingly important therapeutic goals in contemporary CML management. Combining clinical, molecular and comorbidity risk models may help to enable more personalised treatment and optimise long-term outcomes.

## 5 Conclusion

Overall, the rates of hematologic, cytogenetic, and molecular response are high with BCR-ABL tyrosine kinase inhibitors, with excellent disease control and survival in this real-world cohort of patients with chronic myeloid leukaemia. Second-generation TKIs yielded more molecular responses than imatinib. However, there was no difference in survival between the two treatments, as the survival advantage depended primarily on disease biology and the attainment of molecular milestones, not on the first TKI used. Treatment resistance and lack of major molecular response were the most important adverse prognostic factors. Overall, these results support the importance of molecular monitoring, risk-stratified therapy, and tailored treatment strategies in achieving optimal long-term outcomes for CML patients. The finite period of follow-up in the current studies precludes definitive evaluation of late-onset TKI toxicities and very long-term treatment outcomes; however, longer multicentre follow-up, with appropriate molecular and genetic characterisation, is necessary to confirm the long-term safety and durability of these observations.

## Data Availability

The raw data supporting the conclusions of this article will be made available by the authors, without undue reservation.
