# Supplementary material for: Treatment response and survival outcomes with BCR-ABL tyrosine kinase inhibitors in chronic myeloid leukemia: a retrospective study
Source: Front Med (Lausanne). 2026 Aug 19;13:1905107. doi: 10.3389/fmed.2026.1905107 (PMC13533985; doi:10.3389/fmed.2026.1905107)
Supplement: Supplementary file 1 [file Table_1.DOCX]

**Supplementary Table S1. Timing, Reasons for Treatment Switching, Subsequent TKI Selection and Outcomes Among Patients Who Changed Therapy (n = 146)**

| **Variable** | **n (%) or Median (IQR)** |
| --- | --- |
| **Timing of treatment switching** |  |
| Median time to first treatment switch (months) | 13.4 (8.1–19.7) |
| Switched within 12 months | 61 (41.8) |
| Switched after 12 months | 85 (58.2) |
| **Reason for treatment switch** |  |
| Primary resistance | 48 (32.9) |
| Secondary resistance/loss of response | 31 (21.2) |
| Drug intolerance | 53 (36.3) |
| Disease progression | 9 (6.2) |
| Physician/patient preference | 5 (3.4) |
| **Total switched patients** | **146 (100.0)** |
| **Subsequent TKI after switching** |  |
| Nilotinib | 49 (33.6) |
| Dasatinib | 43 (29.5) |
| Bosutinib | 29 (19.9) |
| Ponatinib | 16 (11.0) |
| Other TKI regimens | 9 (6.2) |
| **Total switched patients** | **146 (100.0)** |
| **Clinical outcomes after treatment switching** |  |
| Achieved Complete Cytogenetic Response (CCyR) | 109 (74.7) |
| Achieved Major Molecular Response (MMR) | 96 (65.8) |
| Achieved Deep Molecular Response (DMR) | 56 (38.4) |
| Persistent treatment resistance | 24 (16.4) |
| Required third-line TKI therapy | 28 (19.2) |

Treatment changes were derived from clinical notes and physician notes. Percentages are calculated among patients who had at least one TKI switch during follow up (n = 146). Secondary resistance is the lack of hematologic, cytogenetic or molecular response to a previous treatment. Other TKI regimens" refers to other treatments that are not used as often or to personalized treatments.

**Supplementary Table S2. Baseline Characteristics According to Initial Tyrosine Kinase Inhibitor**

| **Variable** | **Imatinib (n=358)** | **2G TKI (n=292)** | **p-value** |
| --- | --- | --- | --- |
| Age (years) | 51.2 ± 13.9 | 48.1 ± 14.4 | 0.018 |
| Male | 204 (57.0) | 172 (58.9) | 0.63 |
| ECOG ≥2 | 47 (13.1) | 31 (10.6) | 0.34 |
| Chronic phase | 323 (90.2) | 269 (92.1) | 0.42 |
| High ELTS risk | 89 (24.9) | 39 (13.4) | <0.001 |
| Charlson ≥2 | 82 (22.9) | 56 (19.2) | 0.28 |
| Splenomegaly | 228 (63.7) | 173 (59.2) | 0.24 |
| Median WBC | ... | ... | ... |
